# In situ 4D tomography image analysis framework to follow sintering within 3D‐printed glass scaffolds

**DOI:** 10.1111/jace.18182

**Published:** 2021-11-03

**Authors:** Achintha I. Kondarage, Gowsihan Poologasundarampillai, Amy Nommeots‐Nomm, Peter D. Lee, Thilina D. Lalitharatne, Nuwan D. Nanayakkara, Julian R. Jones, Angelo Karunaratne

**Affiliations:** ^1^ Department of Mechanical Engineering University of Moratuwa Moratuwa Sri Lanka; ^2^ Department of Materials Imperial College London London UK; ^3^ School of Dentistry, Institute of Clinical Sciences University of Birmingham Birmingham UK; ^4^ Department of Mechanical Engineering University College London London UK; ^5^ Research Complex at Harwell Didcot UK; ^6^ Dyson School of Design Engineering Imperial College London London UK; ^7^ Department of Electronic and Telecommunication Engineering University of Moratuwa Moratuwa Sri Lanka

**Keywords:** bioactive glass, bioceramics, image analysis, sintering, X‐ray computed tomography

## Abstract

We propose a novel image analysis framework to automate analysis of X‐ray microtomography images of sintering ceramics and glasses, using open‐source toolkits and machine learning. Additive manufacturing (AM) of glasses and ceramics usually requires sintering of green bodies. Sintering causes shrinkage, which presents a challenge for controlling the metrology of the final architecture. Therefore, being able to monitor sintering in 3D over time (termed 4D) is important when developing new porous ceramics or glasses. Synchrotron X‐ray tomographic imaging allows in situ, real‐time capture of the sintering process at both micro and macro scales using a furnace rig, facilitating 4D quantitative analysis of the process. The proposed image analysis framework is capable of tracking and quantifying the densification of glass or ceramic particles within multiple volumes of interest (VOIs) along with structural changes over time using 4D image data. The framework is demonstrated by 4D quantitative analysis of bioactive glass ICIE16 within a 3D‐printed scaffold. Here, densification of glass particles within 3 VOIs were tracked and quantified along with diameter change of struts and interstrut pore size over the 3D image series, delivering new insights on the sintering mechanism of ICIE16 bioactive glass particles in both micro and macro scales.

## 1 INTRODUCTION

Additive manufacturing (AM) allows the fabrication of customized porous architectures with complex geometries and interconnected pores. This enhances the capabilities of porous biomaterials in the field of regenerative medicine, facilitating the development of architectures that cannot be produced by other methods or even patient‐specific implants.^1^ Bioactive ceramics and glasses are promising for bone scaffolds due to their ability to bond with bone.^2^, ^3^ Pore architecture plays a key role in promotion of vascularized bone ingrowth into the scaffold.^4^, ^5^ Conventional methods of producing porous ceramics such as freeze casting,^6^ foaming,^7^ or foam reticulation provide a limited control over pore‐architecture.^8^ With additive manufacturing techniques, pore‐architectures can be predesigned using computer aided design (CAD), providing a better control over pore architecture.^9^ Various AM technologies have been used to fabricate porous ceramic structures, including selective laser sintering (SLS),^10^, ^11^ selective laser melting (SLM),^12^, ^13^ stereolithography (SLA),^14^, ^15^ fused deposition modeling (FDM),^16^, ^17^, ^18^, ^19^ inkjet printing (IJP),^20^, ^21^ binder‐based 3D‐printing (3DP),^22^, ^23^, ^24^ and Direct Ink Writing (DIW).^25^, ^26^, ^27^ In most of these techniques, ceramic particles mixed with a binder are printed into a green body scaffold and then sintered to obtain a fully densified scaffold, which also results in shrinkage of the structure.^1^, ^22^, ^26^

Even though AM can provide exceptional control over architecture, notable structural defects and discrepancies can occur between the final product and the design file. Sintering is an example of postprocessing that contributes to this discrepancy. The aim here was to enable a method of following the changes of particles such as rounding, coalescence, coarsening and residual pores across a 3D‐printed scaffold during sintering and its influence on scaffold's structure at macro scale.

Hot stage microscopy (HSM), dynamic scanning calorimetry (DSC), and differential thermal analysis (DTA) are commonly used to understand sintering of glasses and ceramics.^28^, ^29^, ^30^, ^31^, ^32^ HSM provides information on particle densification with respect to reduction of surface area during sintering,^30^, ^31^ and DTA and DSC provide information on glass transition and crystallization kinetics.^28^, ^31^ Combination of HSM with DSC or DTA deliver a good understanding on sintering kinetics of glasses.^31^, ^32^, ^33^, ^34^ Furthermore, recently Hmood et al. presented that a 3D‐printed pyrometric cone can be used for in situ monitoring of viscous flow and sintering‐crystallization of glass ceramic systems, during heat treatments in a hot stage microscope.^35^ In situ micro‐CT is an effective technique to analyze sintering within ceramic or glass structures, as it provides information on 3D morphological and structural changes occur during the sintering.^36^, ^37^, ^38^, ^39^, ^40^ Nommeots‐Nomm et al. presented 4D analysis of bioactive glass sintering within a 3D‐printed scaffold for the first time. The study delivered new insights on the viscous flow sintering of angular glass particles with a range of sizes. It also revealed a global scale sintering due to the coarsening of the individual struts, emphasizing the importance of evaluating sintering within a 3D‐printed scaffold across the length scales.^41^

While 4D in situ microcomputed tomography (micro‐CT) can be used to capture the sintering process, quantifying these micro‐ and macro‐structural changes requires automated image analysis pipelines. In literature automated thresholding algorithms are widely used to segment the solid phases in micro‐CT images.^39^, ^41^, ^42^, ^43^, ^44^ Thresholding‐based segmentation algorithms, such as Otsu's thresholding, can be accurate in low noise micro‐CT images, where mutually exclusive intensity level distribution can be obtained for solid phase and void. Imaging and reconstruction of a fast dynamic processes such as sintering of angular particles may generate more noise. Image segmentation conducted by thresholding is solely based on the intensity level of each pixel in the image and highly affected by noise. In such cases, advanced segmentation methodologies are required to segment the solid phase of the images. While accurate segmentation of solid phase is a key image processing challenge, implementation of an automated image analysis pipeline for the quantitative analysis of sintering within a 3D‐printed scaffolds in both micro‐ and mesoscale produce more challenges including tracking VOIs and accurate detection of strut boundaries. In the study by Nommeots‐Nomm et al., the quantitative image analysis was conducted based on a 3D image analysis pipeline implemented using Avizo 9.1.1 (FEI Company) and ImageJ. Due to the challenges of automating the pipeline over the complete image series, analysis was conducted with a limited set of scans and VOIs.^41^ Reducing the image series causes information loss and limiting VOIs reduces the accuracy of the quantification. Furthermore, manual selection of VOIs and quantification of descriptors in 3D image sets is tiresome and limits reproducibility.

To resolve this problem, we propose a new automated image analysis framework to analyze micro‐ and macrostructural changes that occur in AM bioceramic scaffolds using 4D image data solving image analysis challenges such as tracking VOIs and accurate segmentation of glassy phase. This image analysis framework incorporates machine learning and was developed using open‐source tools. The proposed image analysis framework will allow studies to investigate dynamic processes that occur within these 3D‐printed bioactive glass scaffolds and optimization of the AM process. The image analysis framework is also applicable in analyzing a wide range of sintering processes.^39^, ^45^, ^46^, ^47^

To demonstrate the image analysis framework, the bioactive glass particles of the composition of ICIE16 (49.46 mol.% SiO_2_, 36.27 mol.% CaO, 6.6 mol.% Na_2_O, 1.07 mol.% P_2_O_5_, and 6.6 mol.% K_2_O) were chosen as it is a highly bioactive glass that can be fabricated into amorphous porous scaffolds using AM techniques such as DIW.^26^ Commercially available bioactive glasses such as the 45S5 Bioglass^®^ and S53P4 tend to crystallize on sintering, so are not appropriate for producing glass scaffolds. Moreover, ICIE16 composition shows promising osteogenic properties with respect to crystallized 45S5 Bioglass^®^.^48^

This is the first 4D analysis of ICIE16 bioactive glass sintering conducted using synchrotron sourced X‐ray computed tomography. Previous study on four‐dimensional quantitative analysis of sintering within a 3D‐printed bioactive glass by Nommets‐Nomm et al. was conducted for the 13–93 (54.6 SiO_2_, 22.1 CaO, 6.0 Na_2_O, 1.7 P_2_O_5_, 7.9 K_2_O, and 7.7 MgO, in mol%) composition.^41^ The 13–93 composition has a higher network connectivity (mean number of bridging oxygen bonds per silicon atom) than 45S5 Bioglass, which should mean that 13–93 is less bioactive than 45S5 in terms of dissolution rate and apatite formation. Here, ICIE16 is of interest as it has a similar network connectivity to 45S5 but can be sintered without crystallization as the temperature difference between the glass transition temperature and the crystallization temperature is sufficient for sintering.^26^, ^41^

In this study, quantitative analysis conducted using the implemented image processing pipeline delivers new insights about the sintering of ICIE16 bioactive glass related to particle densification, sintering stages, shrinkage, and the morphological changes happening within strut of 3D‐printed scaffolds during sintering. Importantly, the proposed image analysis framework facilitates implementing automated, validated, and reproducible pipelines to analyze 3D‐printed porous glasses and ceramics in 4D.

## 2 EXPERIMENTAL PROCEDURE

### 2.1 Scaffold preparation

ICIE16 bioactive glass scaffolds were prepared using the protocol presented by Nommeots‐Nomm et al.^26^ Glass particles were manufactured via melt quenching using high purity silica (SiO_2_) (High Purity, Prince Minerals, Stroke‐on‐Trent), phosphorous pentoxide (P_2_O_5_), and the carbonate equivalent of the required modifying oxides. The glass was melted at 1400°C for 2 h, in a 95% platinum 5% gold crucible, quenched into deionized water, and dried at 100°C. Glass frit was ground in a Ball mill to produce particles with a size distribution ranging from 3.3 to 30.5 μm and *D*_50 _= 10.8 μm was used (measured by a Malvern Mastersizer 2000, Malvern Instruments Ltd. UK). Inks for 3D printing were produced with a 25 wt% Pluronic F‐127 solution (CAS: 9003‐11‐6) and mixed with glass particles (47.5 vol% glass to a Pluronic solution, calculated using the relative glass densities) using a Thinky ARE‐100 mixer until homogenized. Finally, scaffolds were printed using DIW, by a 3D Robocaster (RoboCAD 3.0, 3‐D Inks, Stillwater) with a 250 μm diameter conical nozzle, during the printing a z layer spacing of 200 μm was used (80% of the nozzle diameter). It was found in previous work that 80% is key to insuring layer‐on‐layer adhesion of struts, and adherence of the first layer to the printing substrate.^26^, ^49^ During the printing, the humidity was controlled at 60–80% with a 23°C temperature. Prior to sintering, the size of the printed green body scaffold was 1.8 mm *×* 1.8 mm *×* 1.8 mm.

### 2.2 Imaging and image reconstruction

Viscous flow sintering of the glass particles within 3D‐printed scaffolds were imaged in situ using synchrotron sourced X‐ray computed tomography at the Diamond‐Manchester Imaging Branchline I13‐2 of Diamond Light Source. The optimum sintering temperature for ICIE16 was fixed at 690°C based on previous studies carried out for similar particle sizes.^26^ Sintering was performed in the bespoke proportional‐integral‐derivative‐controlled “Laura” furnace.^41^, ^50^, ^51^, ^52^, ^53^ Here, the glass scaffolds were glued on an alumina sample holder using a high‐temperature glue (OMEGABOND 600; Omega LTD, UK) and alumina holder was mounted on to a rotating spindle on the sample stage. Sintering was carried out in two phases: printed samples were heated up to 500°C at a rate of 3°C per minute and held at 500°C for a dwell 1 h to ensure complete removal of binder (Phase I); then samples were heated at a rate of 3°C per minute up to the sintering temperature 690°C and held for 2 h (Phase II). Any remaining binder would be removed quickly during the ramp up to the sintering temperature. Images were captured throughout the sintering process, and those captured during Phase II were analyzed in this study. The X‐ray microtomography was performed with a filtered pink polychromatic beam in the energy range of 8 to 30 keV. The emitted beam was captured using a CMOS detector with a resolution of 2560*×*2160, which was positioned 75 mm behind the sample stage. All scans were performed with a total magnification of 8*×*, resulting in an effective isotropic pixel size of 0.81 μm. With this setting, projections were taken with an angular step size of 0.09 (2001 projections per 180) for 70 min from the start of Phase II of the sintering, and the rest of the projections were acquired with an angular step size of 0.15° (1201 projections per 180°). Projections were acquired with an exposure time of 45 ms. All the projections were reconstructed using filtered back projection (FBP), incorporating dark and flat field correction and ring artifact suppression.

### 2.3 Development of image analysis framework

The image analysis framework was developed using ImageJ API and Skimage, Sklearn python libraries. The complete set of python scripts for the image analysis framework is available in the provided online repository (https://github.com/AchinthaIroshan/Sintering‐within‐3D‐printed‐ceramics). An image analysis pipeline was implemented using the proposed framework to understand the sintering of ICIE16 bioactive within a 3D‐printed scaffold. Here, 93 3D‐reconstructed images captured during the sintering process were analyzed.

#### 2.3.1 Preprocessing, tracking volumes of interest (VOIs), and denoising of images

**FIGURE 1:**
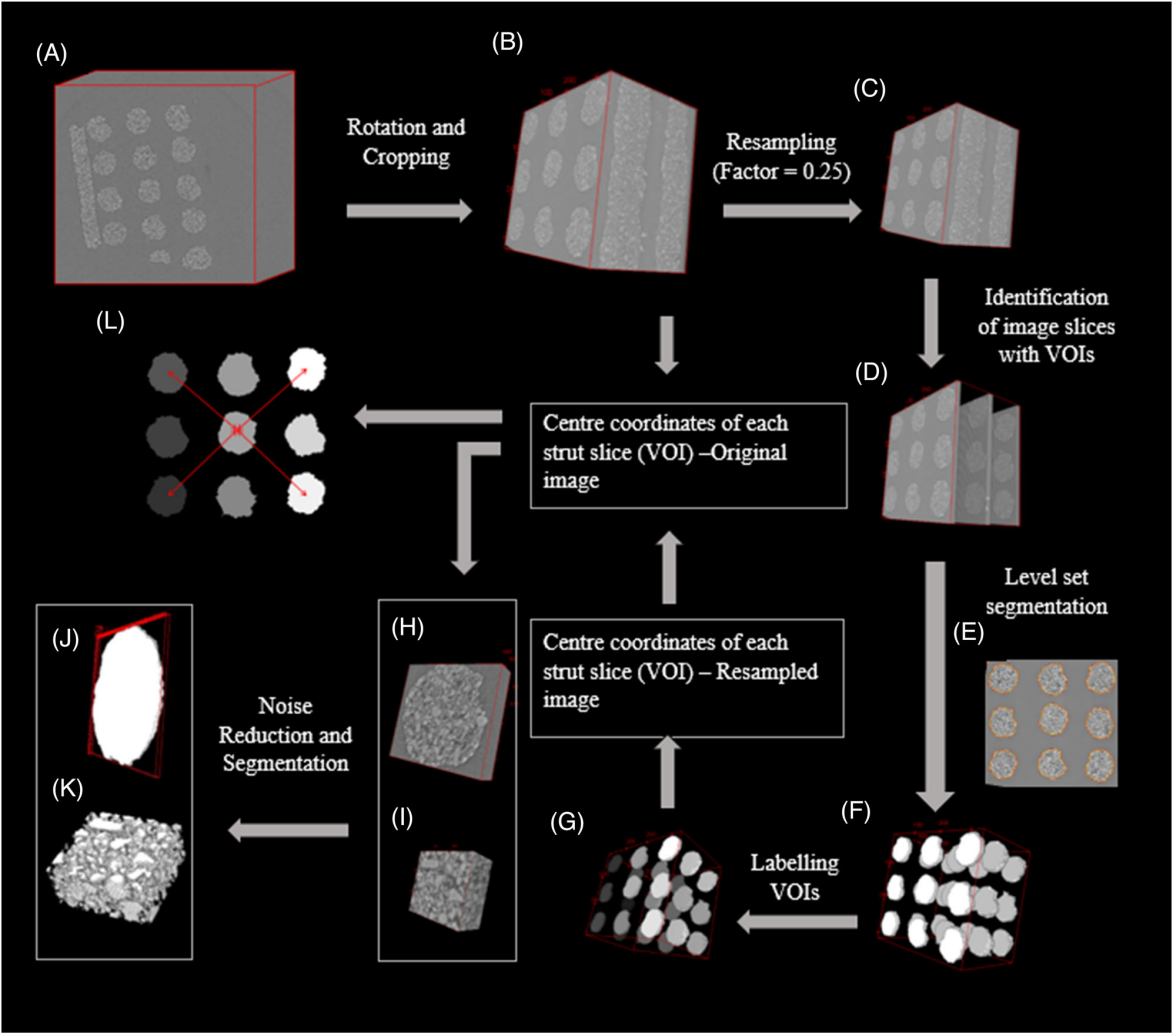
Automated image analysis pipeline to analyze the sintering process. (A) 3D reconstructed image (image size: 2560 × 2560 × 2159). (B) 3D image after initial preprocessing (image size: 1356 × 1296 × 1540). (C) Resampled image (image size: 339 × 324 × 385). (D) Image slices that contain VOIs. (E) Implicit contour to region of interest in 2D. (F) Segmented VOIs (strut slices) in the resampled image. (G) Labeled VOIs (strut slices) in the resampled image. (H) VOI considered to calculate the diameter of a strut. (I) VOI considered to calculated relative densification. (J) Segmented VOI that considered to calculate the diameter of a strut. (K) Segmented VOI considered to calculated relative densification. (L) Distance between strut centers diagonally in the *XY* plane

**FIGURE 2:**
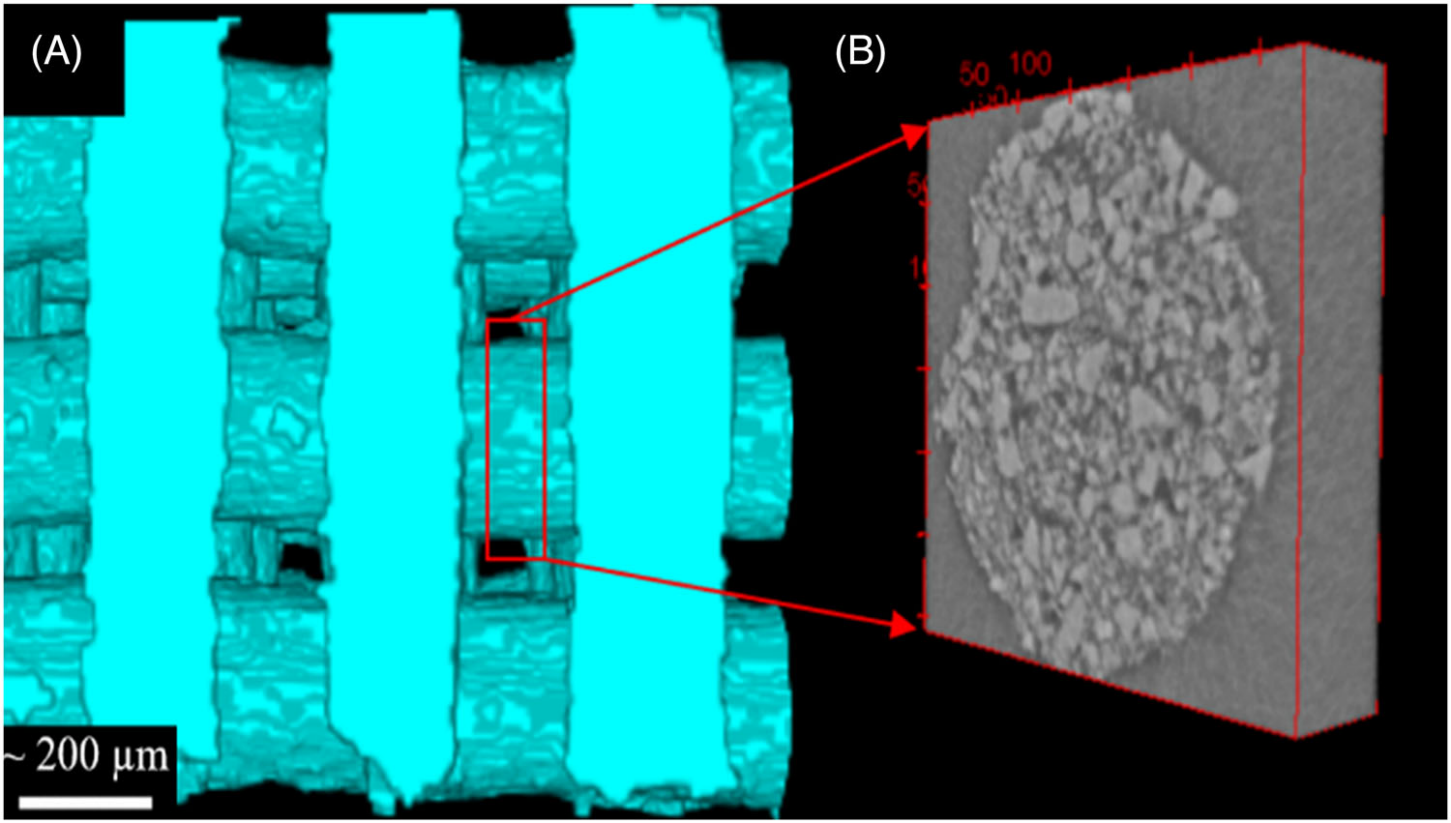
(A) segmented micro‐CT image of a Direct Ink Write bioactive glass scaffold (before the second stage of sintering). (B) Volume of Interest considered in analyzing the sintering of glass particles (slice of a strut)

Initially, all the slices of 3D‐reconstructed images (example: Figure 1A) were converted to 8 bits. Then initial preprocessing was conducted by rotating and cropping each 3D image (Figure 1B). Here, the rotation was conducted such that the struts of the scaffolds were aligned with *y* and *z* directions of the image. Struts can be considered as the building element of the scaffold (Figure 2). Therefore, cropped 3D slices of struts with a thickness of 57.6 μm (example: Figure 2B) were taken as volumes of interest (VOIs) to analyze sintering.

VOIs of each image was detected using an algorithm propose with the image analysis framework. Here, the VOIs were detected by automated identification of centers of those volumes (centers of selected strut slices). The main steps of the developed algorithm are described below.

- **Resampling of the 3D image with a factor of 0.25 (**Figure 1C): Here the size of the image is reduced to 1/4th of the original size, which makes it easier to compute macroscale features to detect VOIs.
- **Detecting image slices, which include VOIs using a Support Vector Machine (SVM) classifier** ^54^ (Figure 1D): The 3D image can be represented as a stack of 2D images where the 2D image is called as an image slice. A set of image slices labeled with two classes (Class 1—Slices that include VOIs, Class 2—Slices that do not include VOIs) were used as training data to train a Support Vector Machine classifier, which is a machine learning classification model. The trained model was used to detect slices that include VOIs in each 3D image.
- **Segmenting regions with strut cross‐sections in each detected slice using “Level‐set” algorithm** ^55^ (Figure 1E, F): In “level‐set” segmentation algorithm, an implicit contour is fitted to boundaries of regions of interest (Figure 2E)
- **Labeling each connected component in the 3D image** (Figure 1G): Here, each connected component is considered as a VOI in the resampled 3D image. Each connected component is labeled to obtain their centers separately.
- Calculating the center coordinates of each labeled 3D‐connected component: *regionprops* of Skimage was used to calculate the center.^56^, ^57^
- **Converting the calculated coordinates in coordinates of the original size image**: As volumes of interests in the original images are considered for the analysis, center coordinates of VOIs in the resampled 3D image were converted to the coordinates of the original size image.
- **Obtaining VOIs based on the center coordinate** (Figure 2H, I): For each obtained center coordinate, volumes of dimensions 150 × 140 × 70 (Figure 2H) and volumes of dimensions 150 × 140 × 70 (Figure 2I) were generated at each time point to quantify diameter and densification, respectively.

4D hyperstacks (3D volume series) of each VOI were generated by combining 3D volumes based on Euclidian distance between center coordinates of volumes in consecutive time points. Based on the obtained center information, 27 VOIs can be obtained from each 3D image, and 27 4D hyperstacks can be generated from the complete scan set captured during the sintering. Here 3 VOIs from each 3D image were taken for the analysis.

Nonlocal mean filtering^58^ was applied to further noise reduction, which uses redundant information of the image to reduce the noise by performing a weighted average of pixel values considering spatial and intensity similarities between pixels. Following the noise‐reduction contrast of images was enhanced using histogram equalization.

#### 2.3.2 Segmentation of strut and glassy phase in VOIs

The implemented image analysis pipeline to characterize sintering in the microscale consists of two main segmentation components. One of those components is segmenting the glassy phase within a strut to analyze densification during sintering. A random forest classifier was applied to segment the glass phase until glass densification reached about 90%. The fast random forest classification is a machine learning method, which can be used for image segmentation. In this work, it was implemented incorporating the Trainable Weka Segmentation module of Fiji^59^ and trained to classify every voxel to glassy class or void class. A total of 162 792 labeled voxels were used in training the model, where 96 323 voxels were labeled into pore class and 66 469 into glassy class. During the model training, each labeled voxel was represented using 77 attributes based on Gaussian blur, Hessian matrix, Difference of Gaussian, Sobel filters, and directional filtering. Here, a random forest classifier with 200 trees was constructed. Once the densification of the glassy phase reached around 90%, images were segmented using Otsu's thresholding method.^60^ The second segmentation component of the image analyzing pipeline is segmenting the strut without considering intrastrut porosity to analyze the diameter change of struts during the sintering. Here, a child volume of size 330 × 306 × 10 was obtained from the VOIs of each 3D image without changing the center of the VOI. Then, strut regions of each image slice were segmented using the level‐set algorithm to evaluate the change of strut diameter during the sintering.

The performance of image segmentation was evaluated by calculating the accuracy, precision, and Intersect over Union (IoU) values of each segmentation method. These performance measures were calculated based on a set of manually segmented images that were taken as ground truth segmentations.

#### 2.3.3 Quantitative analysis of ICIE16 bioactive glass sintering

Three parameters were quantified for the quantitative analysis. Those are the relative densification of the glassy phase, change of diameter of the struts, and change of longest diagonal length of the interstrut pores during the sintering. Here, a complete scan set with a size of 93, captured during the second stage of the sintering, was analyzed.

To quantify the relative densification of glass at the time of a 3D scan was acquired, segmented binary volumes of 150 × 140 × 70 (Figure 1J) were obtained from the detected VOIs of 330 × 306 × 70, without changing the center of the volumes. Glass volume and pore volume in each VOI were calculated by counting corresponding voxels. The intrastrut porosity and relative density of the glassy phase were calculated using Equations (1) and (2).

(1) $$Porosity\;\left(\%\right)=\frac{Pore\;Volume}{Pore\;Volume+Glass\;Volume}\;\times 100,$$

(2) $$\begin{array}{c} Relative\;Density\;of\;Glassy\;Phase\;\left(\%\right)=100-Porosity. \end{array}$$

To quantify the diameter of a strut, segmented binary volumes of 330 × 306 × 10 (Figure 1K) were obtained from the detected VOIs of 330 × 306 × 70 without changing the center of the volume. Then the cross‐sectional area of the strut in each image slice was calculated. Here, the number of pixels in the foreground in each slice was taken as the area. The diameter was calculated using the Equation (3), assuming that the cross‐section of a strut is circular.

(3) $$Diameter=2\times \sqrt{\frac{Area}{\pi }}.$$

The interstrut pore diagonal distance (PDD) at each time point of a scan was calculated using Equation (4),

(4) $$\begin{array}{ccc} PDD & = & Distance\;between\;strut\;centers\;diagonaly\\ & & -Diameter\;of\;struts, \end{array}$$

where the distance between strut centers placed in directions of pore diagonals was obtained as illustrated in Figure 1L.

The strut diameter of each VOI was obtained (3 VOIs) and pore diagonal distance was obtained considering 3 interstrut pore channels in each 3D image. The averages of those automated measurements were obtained as an estimator for the strut diameter and pore diagonal distance of the scaffold at the time point of the scan. As the calculation are obtained in pixels, measurements were rounded to the nearest integer and multiplied by 0.81 (the isotropic pixel size) to convert into micrometers.

Finally, strut diameter values, the relative densification of glass phase, and the longest diagonal length of interstrut pores were plotted against temperature and time for the complete 3D image series representing the sintering of ICIE16 bioactive glass within a 3D‐printed scaffold.

## 3 RESULTS AND DISCUSSION

### 3.1 Image analysis framework

The accuracy of 4D image quantification is highly reliant on the image analysis pipeline. Therefore, image analysis plays a vital role in the characterization of dynamic processes using 4D imaging. In this study, the relative density of the glassy phase, the diameter of the strut and interstrut pore diagonal were quantified as descriptors to analyze the sintering within a 3D‐printed scaffold both micro‐ and macroscales.

#### 3.1.1 Detection of VOIs and tracking

Based on the visualization of the series of 93 3D images (Video S1), it was identified that VOIs considered for the analysis were not static over time due to the shrinkage of the scaffold. Manual identification of VOIs in each 3D image is a challenging and time‐consuming task. Therefore, the proposed algorithm to detect and track VOIs is a vital component of the image analysis pipeline. Dynamic visualization of VOIs obtained from the VOI detection and tracking algorithm is included in Video S2.

#### 3.1.2 Noise reduction

**FIGURE 3:**
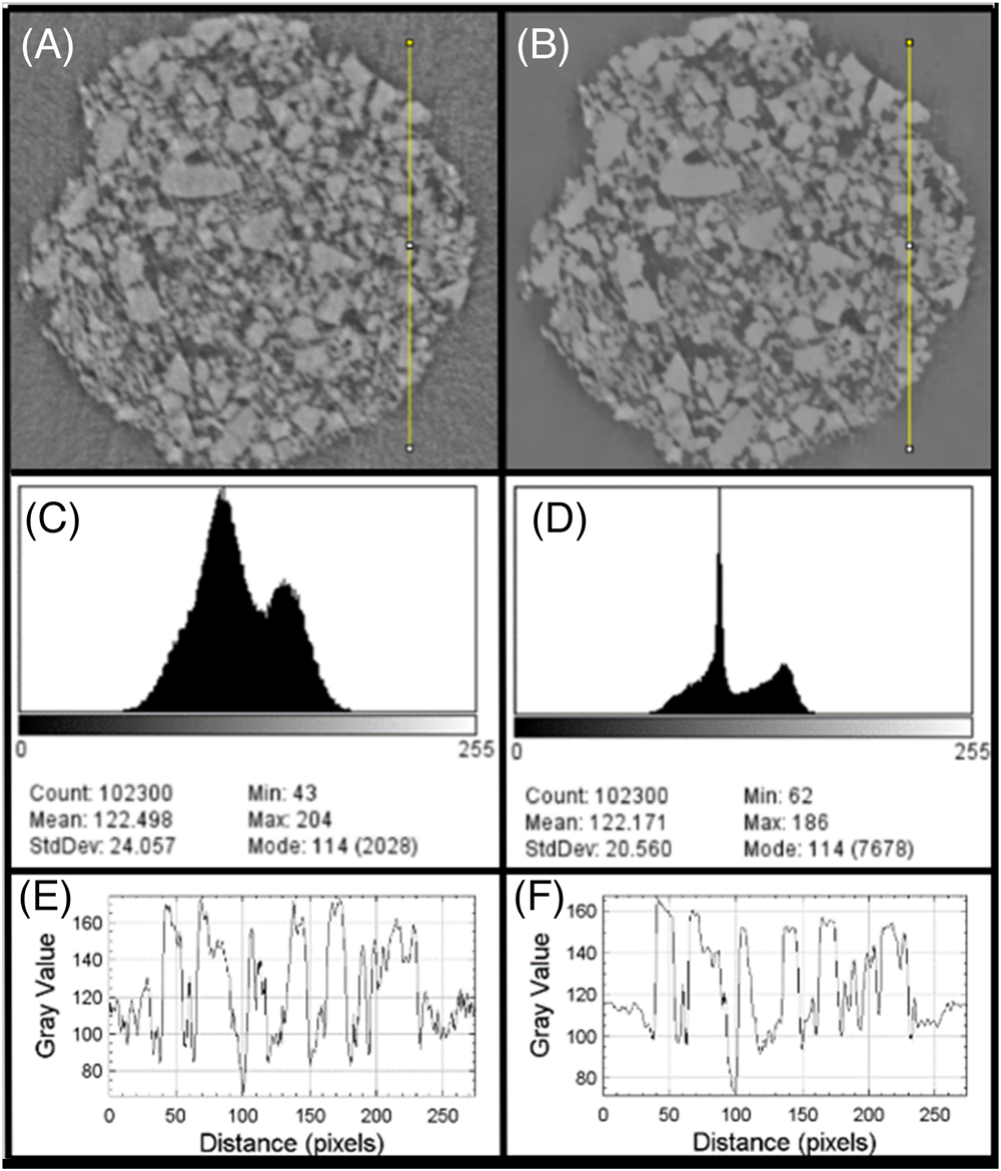
Response of the nonlocal means filter. (A) Cross‐section of a strut—original image. (B) Response of nonlocal means filter. (C), (D) Histograms of (A), (B); (E), (F) line intensity profiles of lines drawn on (A), (B)

Noise reduction of the detected child volume highly affects the accuracy of the image segmentation. Notable noise reduction of VOIs was achieved by applying nonlocal means filter. Resulting images (Figure 3A, B), histograms (Figure 3C, D), and intensity profiles of lines drawn on top of original and resulting images (Figure 3E, F) provide clear evidence of noise reduction. As shown in the histograms (Figure 3C, D), even after the noise reduction, intensity values of glass and pore voxels could not be observed as mutually exclusive sets.

#### 3.1.3 Image segmentation and quantification

**FIGURE 4:**
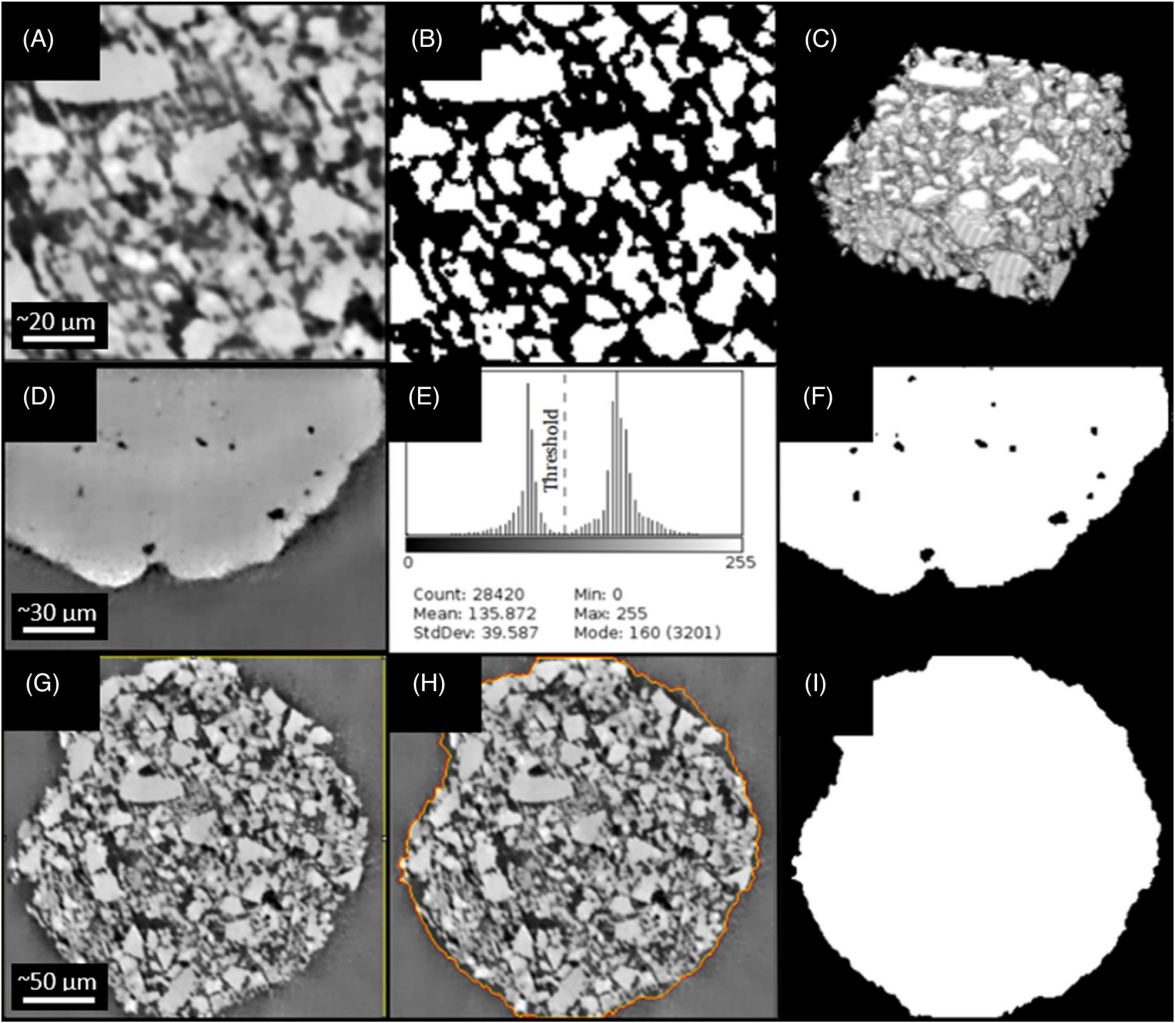
Image segmentation of micro‐CT images. (A) Preprocessed image slice of a VOI obtained to quantify relative densification where individual glass particles are visible. (B) Segmented image of glassy phase using fast random forest voxel classification. (C) Segmented image volume considered to calculate the relative densification of the glassy phase. (D) Section of a preprocessed image captured after reaching a higher densification (around 90%). (E) Histogram of the image (D). (F) Image of the glassy phase segmented using Otsu's thresholding. (G) Preprocessed image slice of a VOI, obtained to quantify diameter change of struts. (H) Contour of level‐set function fitted to the boundaries of the strut cross‐section. (I) Segmented strut cross‐section obtained using the level‐set segmentation

As mutual exclusive intensity distribution was not obtained with the noise reduction, random forest classification, based on machine learning, was used to segment the glassy phase of VOIs. In random forest classification, several image features are used for the image segmentation. The performance Fast Random Forrest classification methods to segment glassy phase was evaluated with respect to Otsu's thresholding method, using intersection over union (IoU), accuracy, and precision (Table 1). Fast random forest delivered a better performance over Otsu's thresholding in segmentation of glassy phase for the images captured within the first 1 h and 15 min of sintering (early sintering stages) where mutually exclusive intensity level distribution was not obtained for glass and void. Figure 4A–C visualizes the results of segmentation obtained with fast random forest classifier. The out‐of‐bag error of the fast random forest classifier was 4.88%. After 1 h and 15 min of sintering, the densification of the glassy phase reached about 90% (Figure 4D). At this stage, mutually exclusive intensity levels can be observed for the glassy phase and void (Figure 4E). Therefore, Otsu's thresholding provided good results for glassy phase segmentation (Figure 4F) of those images with high IoU, accuracy, and precision (Table 1).

**TABLE 1:** Performance evaluation of the image segmentation

Volume of interest	Segmentation algorithm	Intersection over union (IoU)	Accuracy	Precision
Glassy phase within a strut (relative densification ≤90%)	Fast random forest	0.83	0.93	0.91
	Otsu's thresholding	0.78	0.88	0.79
Glassy phase within a strut (Relative densification ≥90%)	Otsu's thresholding	0.98	0.98	0.99
Strut slice, excluding pores within the strut	Level‐set segmentation	0.96	0.97	0.99

To quantify the diameter of struts, boundaries of the struts should be accurately detected. Due to the noise and the intrastrut porosity (Figure 4G), accurate identification of strut boundaries is a challenging task. “Level‐set” segmentation was identified as the most suitable algorithm to detect the boundaries of the strut accurately. “Level‐set” segmentation is carried out by fitting an implicit contour to strut boundaries (Figure 4H), resulting in a good segmentation (Figure 4I) with high IoU, accuracy, and precision (Table 1).

In the implemented image analysis pipeline, the segmentation is followed by the quantification descriptors to analyze sintering. The results of quantification and insight delivered on sintering are discussed in the next section. Furthermore, the presented image analyzing methodologies are not limited to analyzing the sintering within 3D‐printed scaffolds. These techniques can also be used to analyze the structural changes, which occur in scaffolds designed using woodpile architecture and to analyze the densification of different particles within solid structures, incorporating 4D imaging.

### 3.2 Sintering analysis

In this work, changes in relative density of the glassy phase during the sintering were quantified as a descriptor for the microscale analysis of sintering, and both diameter and the interstrut pore diagonal distance were quantified as descriptors for macroscale analysis of sintering.

According to the classical sintering theory on densification, powder compaction is described with three defined stages. During the initial stage of the sintering, neck formation occurs between particles resulting around 10% of densification. During the intermediate stage, bulk densification occurs, collapsing interconnected pores into isolated pores reaching approximately 90% relative densification. During the final stage, size of closed pores reaches toward zero resulting 0% porosity and complete densification.^61^, ^62^

**FIGURE 5:**
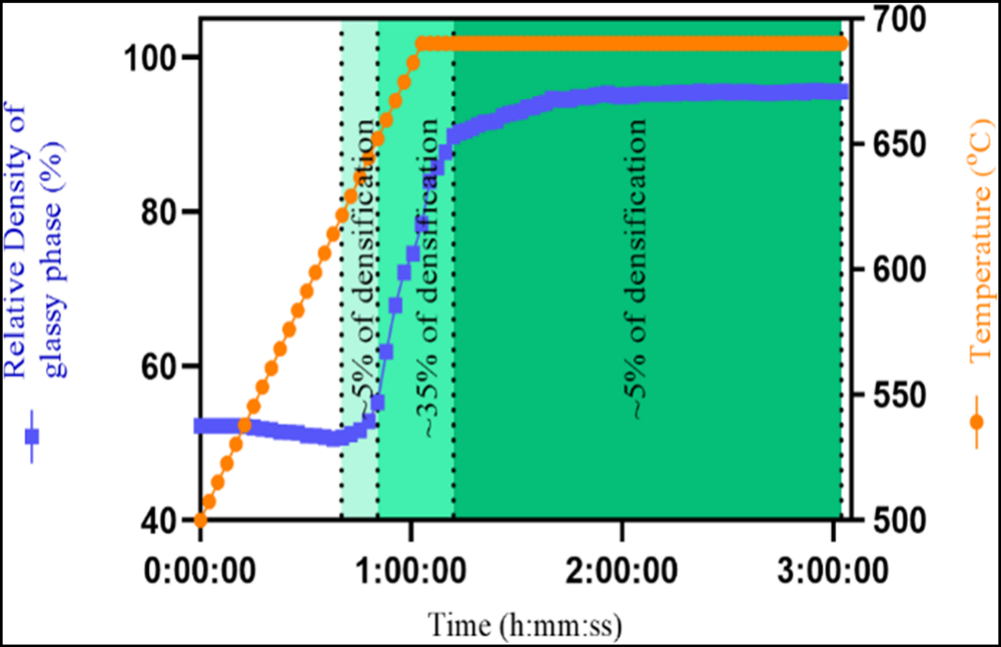
Densification of glass particles with a 3D‐printed scaffold as temperature increased during sintering

**FIGURE 6:**
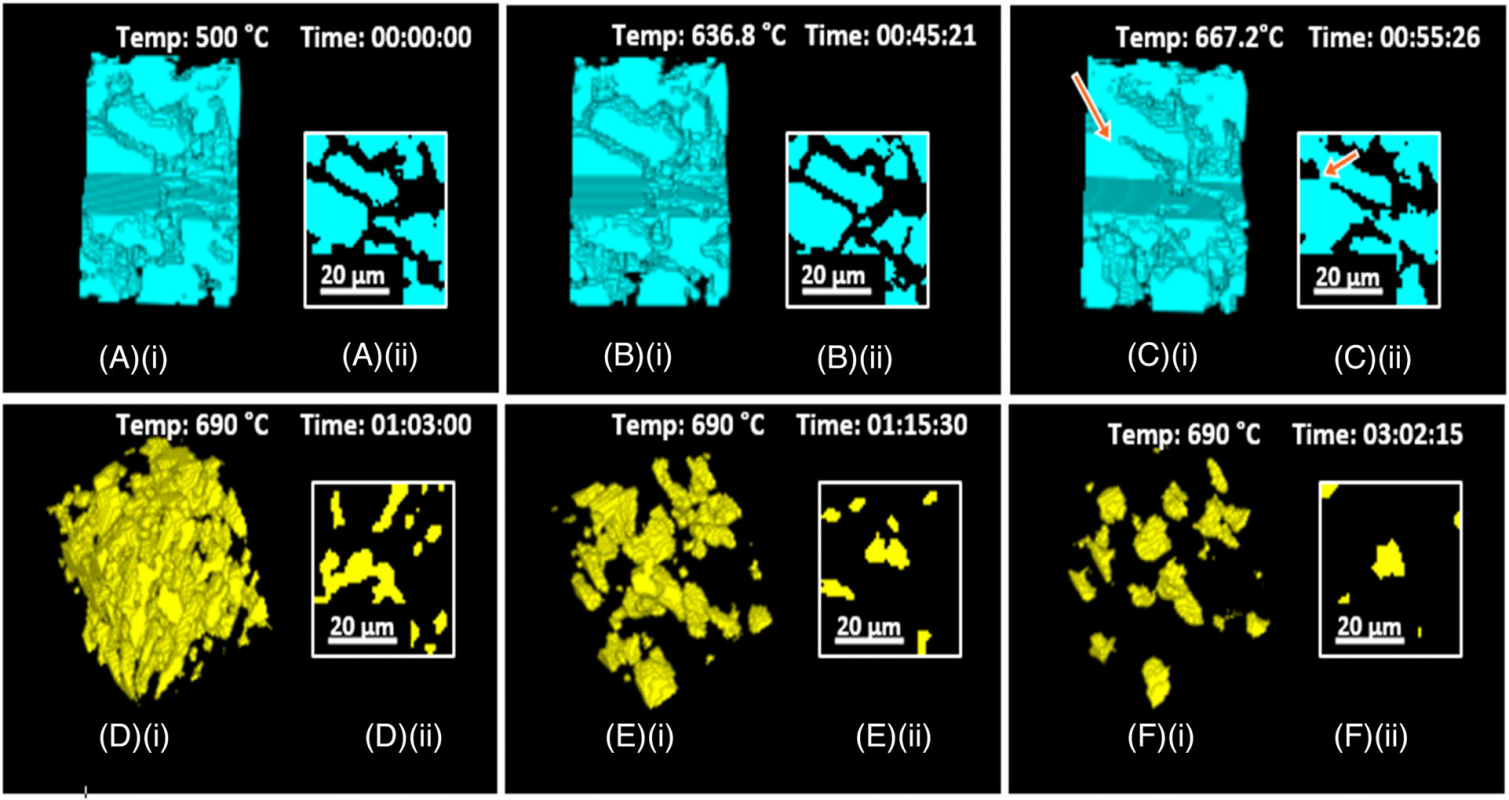
Visualization of child volumes cropped from a VOI of the scaffold obtained from micro‐CT images at different stages of sintering after segmenting the glassy phase and intrastrut pores: (A) (i), (B) (i), (C) (i) morphological changes of glass particles within a child volume of a strut in the early stage of sintering; (D) (i) pore network within a child volume of a strut during the intermediate sintering stage; (E) (i) pores within a child volume of a strut just after the intermediate sintering stage; (F) (i) pores of a child volume within a strut at the end of sintering. (A) (ii), (B) (ii), (C) (ii), (D) (ii), (E) (ii), (F) (ii) show 2D image slices of volumes (A) (i), (B) (i), (C) (i), (D) (i), (E) (i), (F) (i), respectively

The graph (Figure 5) shows the change of relative density of the glassy phase over time and temperature. As presented in the graph, three stages of densification were visible with the ICIE16 bioactive glass sintering cycle, confirming the classical sintering theory. Here, ∼5% of densification occurred during the initial sintering stage, ∼35% of fast densification happened during the intermediate sintering stage, and ∼5% of slow densification during the fast sintering was observed. Furthermore, microstructural changes within a strut were observed by visualization of segmented child volumes of VOIs at different stages of sintering (Figure 6). Closely packed glass particles were observed within struts of the scaffold after the removal of binder at the temperature of 500°C (Figure 6A, Time 00:00:00). No notable morphological changes of glass particles were observed for the first 45 min with gradual increase of temperature to 636.8°C (Figure 6B, Time 00:45:21). Once the temperature reached 667.2°C, neck growth between glass particles was observed (Figure 6C, Time 00:55: 26), which is a feature of the initial stage of sintering according to classical sintering theory. During the intermediate sintering stage, interconnected pores were present (Figure 6D, Time 01:15:21). Within a very short time (12 min), these interconnected pores transformed into isolated pores (Figure 6E, Time 01:15:30). During the final sintering stage, the isolated pores were further reduced. A set of tiny (less than 20 μm in size) isolated pores were observed at the end of the 3‐h sintering cycle (Figure 6F, Time 03:02:15). These observations confirm that the densification of ICIE16 bioactive glass follows classical sintering theory. Nommeots‐Nomm *et al*. previously showed that the sintering of glass particles of the 13–93 composition within a 3D‐printed scaffold also followed classical sintering theory.^41^

**FIGURE 7:**
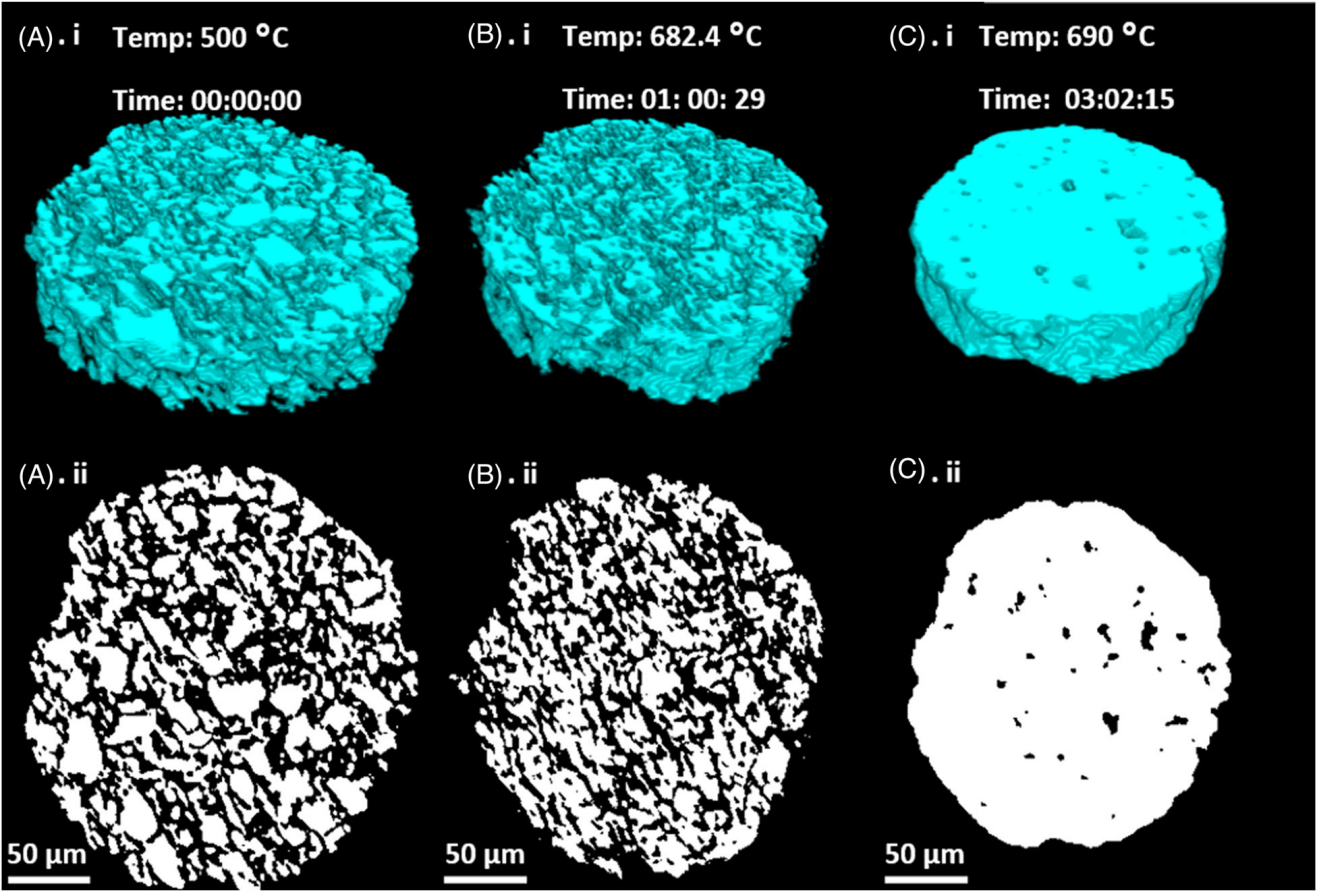
VOIs of micro‐CT images obtained at different stages of sintering: (A) (i) Segmented slice of a strut considered in sintering analysis, captured after the complete burn out of the binder. (B) (i) Segmented strut slice during the fast sintering stage. (C) (i) Segmented strut slice after the completion of the sintering process (A) (ii), (B) (ii), and (C) (ii) are cross‐sections of (A) (i), (B) (i), and (C) (i) sequentially

Figure 7 visualizes changes in a slice or section of a strut (VOI) within the scaffolds during the different sintering stages, which indicate the nature of a strut at the start of the sintering (Figure 7A, Time 00:00:00), showing individual particles and cylindrical morphology of the strut. At the intermediate stage of the sintering (Figure 7B, Time: 01:00:29), shrinkage of the strut occurred, and at the end of sintering (Figure 7C, Time: 03:02:15), a densified strut with a smooth surface was observed at the end of the sintering. Here (Figure 7), a notable reduction of strut diameter (15%) can also be observed.

The glass particles are packed within the strut of the scaffold. If we assume that the shrinkage of the strut due to the sintering of glass particle is isotropic, the relationship between the volume shrinkage and the linear shrinkage is as follows:^63^

(5) $$Volume\;Shrinkage=1-{\left(1-linear\;shrinkage\right)}^{3}.$$

**FIGURE 8:**
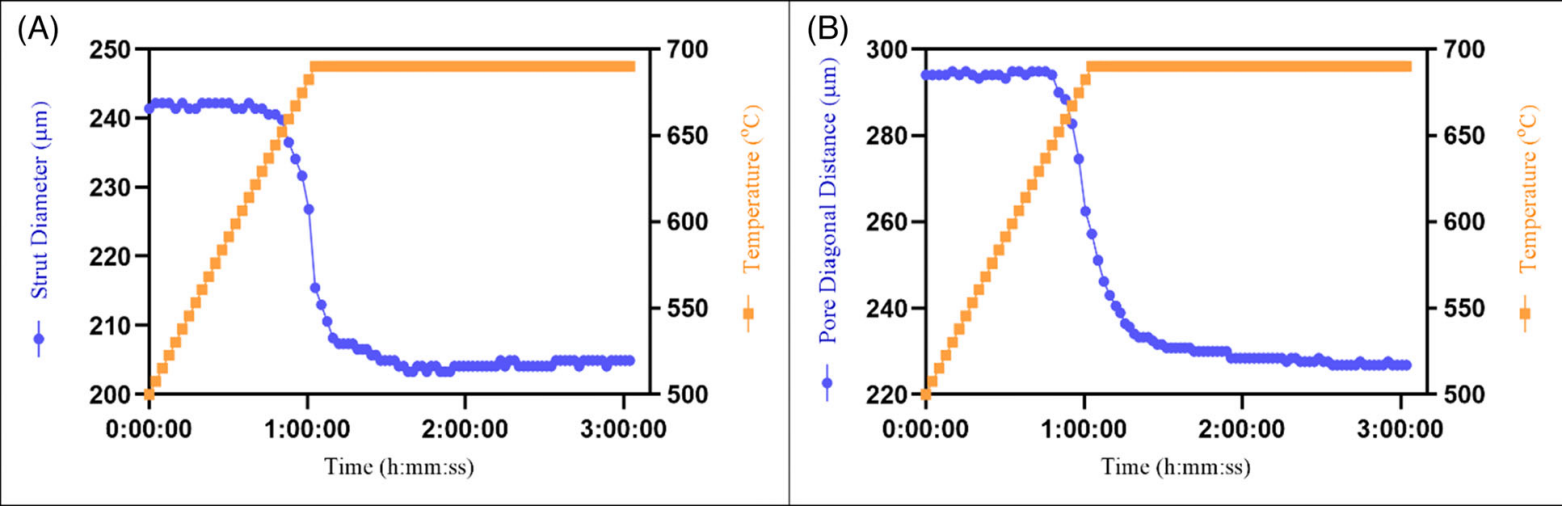
Quantification of changes in the global‐level 3D‐printed scaffold architecture as a function of sintering time, by image analysis: (A) Change of strut diameter. (B) Change of pore diagonal distance

The graph (Figure 8A) shows the change of the strut diameter during the sintering. Initial diameter of a strut was 241.4 μm. Once the temperature reached 660°C, the diameter rapidly reduced from 239.8 to 207.4 μm within 23 min as a result of bulk densification during the intermediate sintering stage. The diameter further reduced at 690°C and reached a minimum of 203.3 μm. The graph shows that the strut diameter remained at 204.1 ± 0.8 μm during the final stages of sintering.

The change of pore diagonal distance also shows a pattern similar diameter reduction and shows a continuous decreasing (Figure 8B). The pore diagonal distance of the printed scaffold was 294.0 μm. Same as with the strut diameter, once the temperature reached 660°C, pore diagonal distance rapidly reduced from 288.4 to 239.0 μm within 23 min. The diagonal distance continues to decrease at 690°C, reaching a minimum of 226.0 μm.

**FIGURE 9:**
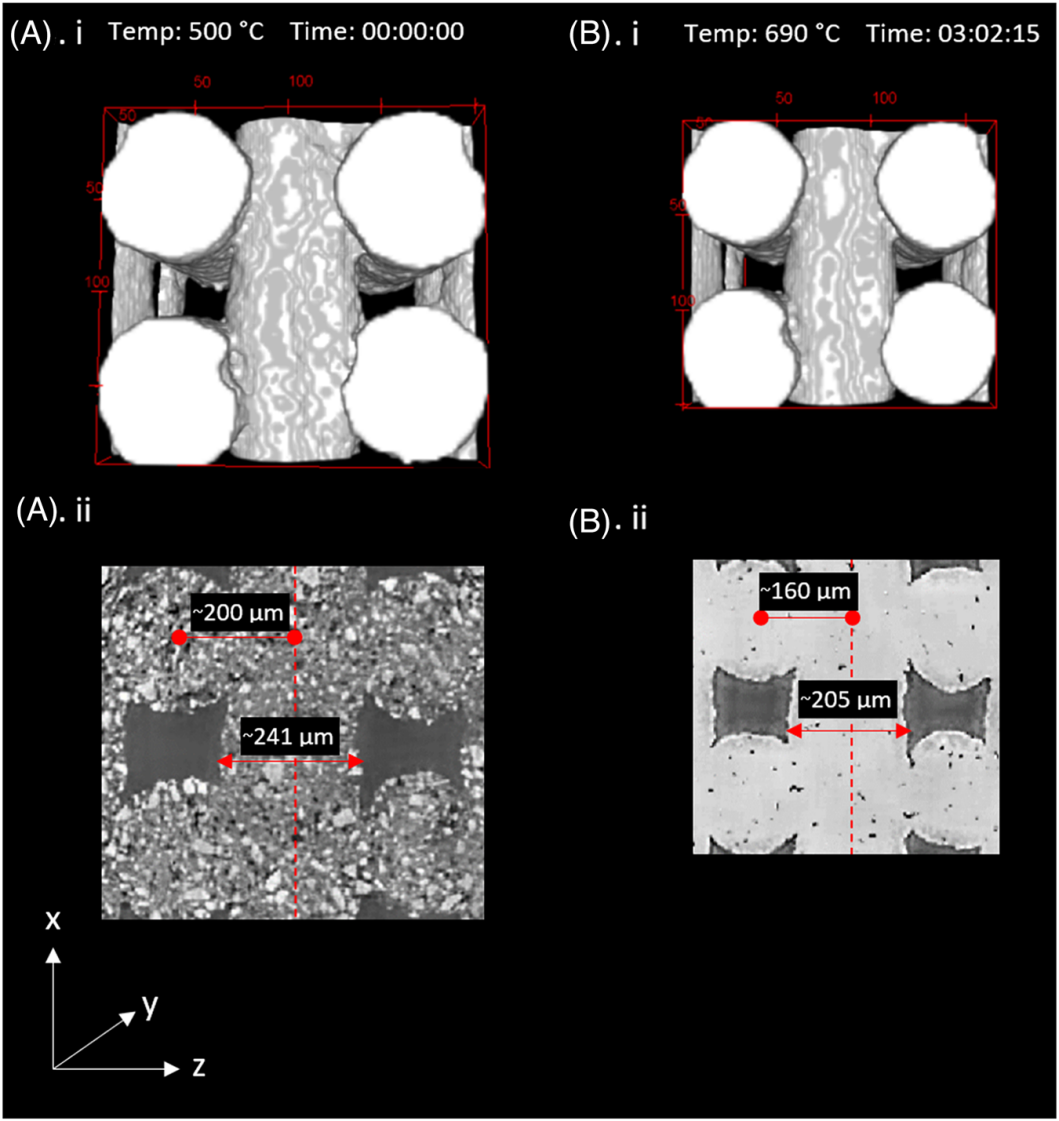
Shrinkage of a child volume within the scaffold after the sintering. (A) (i) Child volume before sintering and (ii) image slice of the child volume (A) (i), which captures centerline of strut layer over *x*‐direction. (B) (i) Child volume after sintering and (ii) image slice of the child volume (B) (i), which captures the centerline of the strut layer over *x*‐direction The bounding box is scaled in pixels where 1 pixel length = 3.21 μm

The reduction of the diameter was 15.8%. If we consider the reduction of the diameter as linear shrinkage, the volume shrinkage can be calculated using Equation 5, giving a value of 40.4%. To evaluate whether the shrinkage of the scaffold was isotropic, volume shrinkage was calculated based on the child volumes shown in Figure 9. The shrinkage of the considered child volume was 46.7%, which proves that the volume shrinkage was not isotropic. This confirms the shrinkage results published by Nommeots‐Nomm et al. using bulk measurements, which showed that linear shrinkage in the *z* direction was less than that of the scaffold in *x* and *y* directions.^26^ To develop patient‐specific scaffolds, the shrinkage should be predetermined and incorporated into initial design. As this analysis suggests that shrinkage is not isotropic, more advanced models should be implemented to predict the shrinkage.

The global scale sintering within a 3D‐printed scaffold occurs due to the coarsening of struts of the scaffold that appears at stage 3 of particle sintering. This was initially observed by Nommeots‐Nomm et al. during the sintering of 13–93 bioactive glass 3D‐printed scaffold. In their work, they identified a steady increase of strut diameter and a steady decrease of the longest pore diagonal length during stage 3 of sintering.^41^ This happened as a result of the coarsening of individual struts, which can be identified as a sintering at a global level. However, this change was not clearly observed (Figure 8) with ICIE16 bioactive glass composition. Furthermore, the *z*‐layer spacing was set as 200 μm in the initial printing parameters and it was maintained in the printed green body scaffold (Figure 9Aii), resulting in a strut overlap of ∼41 μm (∼17% of strut diameter), and it was observed that z‐layer spacing reduced to 160 μm (Figure 9Bii), resulting in a strut overlap of ∼ 49 μm (∼ 21% of strut diameter). The strut overlap was calculated based on the measurement taken from images shown in Figure 9Aii and Bii. This ∼4% decrease of strut overlap is also not a strong indication for the coarsening of struts due to global scale sintering. Yet, we cannot avoid conclude that there is no global scale sintering in ICIE16 bioactive glass.

## 4 CONCLUSIONS

We developed an image analysis framework incorporating machine learning to investigate the viscous sintering of particles with randomly distributed particle size (nonidealized model) within 3D‐printed scaffolds using synchrotron X‐ray tomography. The image analysis framework was validated and able to automate four‐dimensional analysis of sintering within 3D‐printed structures. To demonstrate the developed image analysis framework, we carried out a 4D automated micro‐ and macroscale analysis of ICIE16 bioactive glass sintering within a 3D‐printed scaffold designed for bone tissue engineering for the first time. The densification of bioactive glass particles was quantified during the sintering considering multiple VOIs over time and its influence on scaffold's structure was identified. Shrinkage was found to be anisotropic and the degree of sintering must be included in the 3D design files for the scaffolds in the future, especially for patient‐specific scaffolds. Sintering of the ICIE16 composition follows classical sintering theory and left some residual spherical pores within the struts. The findings of this research will be helpful in the implementation of a computational model to predict the shrinkage of bioactive glass sintering, which will enable producing patient‐specific scaffolds with designable geometries. Furthermore, the proposed image analysis framework is not limited to synchrotron tomography data and can be applied with other 4D imaging modalities which can capture microscale dynamics.

## Supporting information



Supporting Information

Supporting Information
