# Supplementary figures and images for: In situ 4D tomography image analysis framework to follow sintering within 3D‐printed glass scaffolds
Source: J Am Ceram Soc. 2021 Nov 3;105(3):1671–84. doi: 10.1111/jace.18182 (PMC9297994; doi:10.1111/jace.18182)

## Slide 1
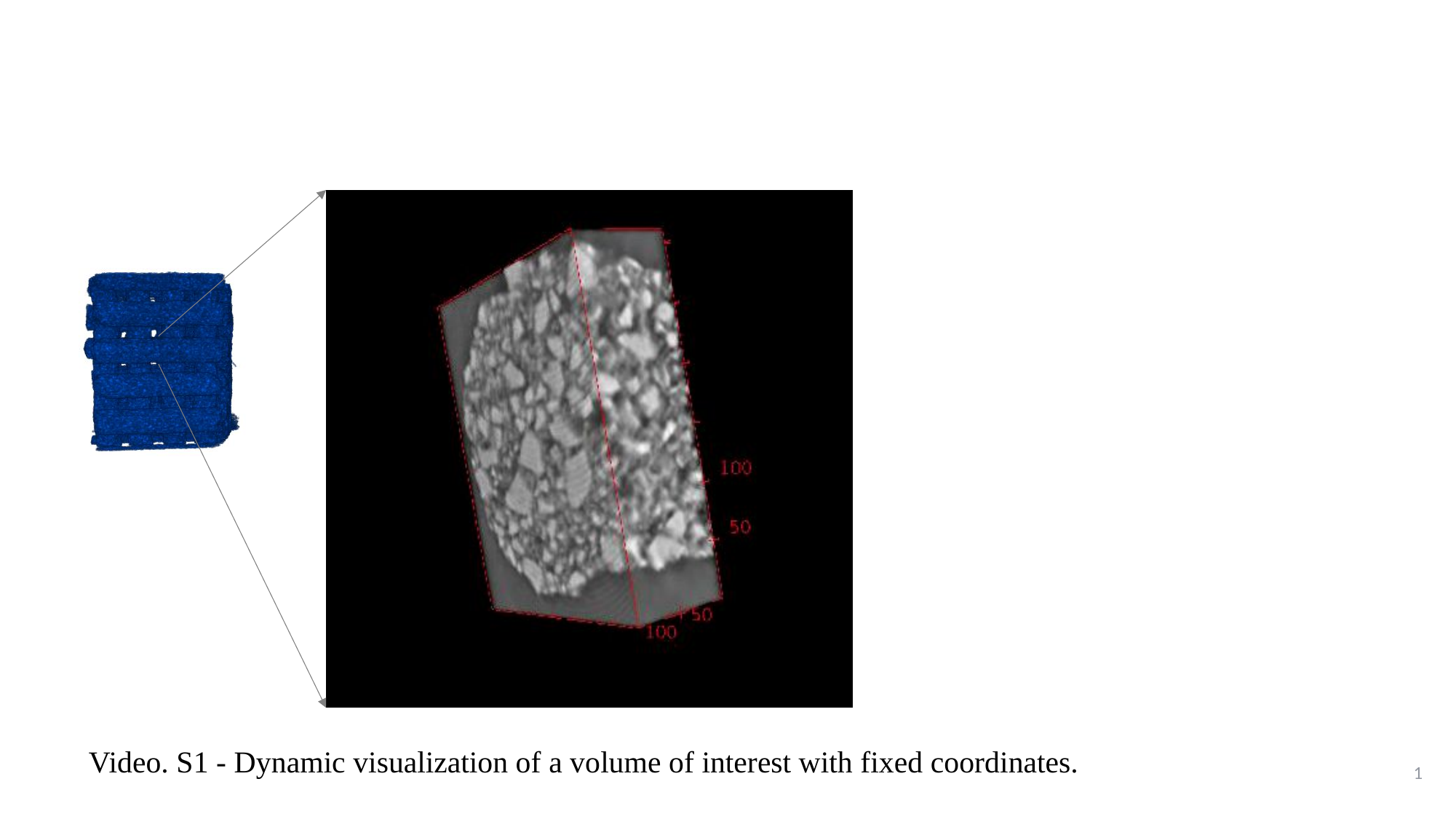

# Video. S1 - Dynamic visualization of a volume of interest with fixed coordinates.
1

Supplement: Supplementary file 1 — Supporting Information [file JACE-105-1671-s001.pptx]
